# Influence of Water Content on Mechanical Strength and Microstructure of Alkali-Activated Fly Ash/GGBFS Mortars Cured at Cold and Polar Regions

**DOI:** 10.3390/ma13010138

**Published:** 2019-12-29

**Authors:** Xiaobin Wei, Feng Ming, Dongqing Li, Lei Chen, Yuhang Liu

**Affiliations:** 1 State Key Laboratory of Frozen Soil Engineering, Northwest Institute of Eco-Environment and Resources, Chinese Academy of Sciences, Lanzhou 730000, China; 2 University of Chinese Academy of Sciences, Beijing 100049, China

**Keywords:** geopolymer, water content, negative temperature, compressive strength, microstructure, elastic modulus

## Abstract

Negative temperature curing is a very harmful factor for geopolymer mortar or concrete, which will decrease the strength and durability. The water in the geopolymer mixture may be frozen into ice, and the water content is a crucial factor. The purpose of this paper is to explore the influence of water content on the properties of alkali-activated binders mortar cured at −5 °C. Fly ash (FA) and ground granulated blast furnace slag (GGBFS) were used as binders. Three groups of experiments with different water content were carried out. The prepared samples were investigated through uniaxial compression strength test, Scanning electron microscopy (SEM), and X-ray diffraction (XRD) for the determination of their compressive strength, microstructural features, phase, and composition. The results indicated that, the compressive strength of samples basically maintained 25.78 MPa–27.10 MPa at an age of 28 days; for 90 days, the values reached 33.4 MPa–34.04 MPa. The results showed that lower water content is beneficial to improving the early strength of mortar at −5 °C curing condition, while it has little impact on long-term strength. These results may provide references for the design and construction of geopolymer concrete in cold regions.

## 1. Introduction

Production process of ordinary Portland cement (OPC) not only consumes plenty of energy and natural resources, but also emits large quantities of carbon dioxide [1,2,3]. A host of researchers have done abundant work in search of an environmentally friendly alternative binding material. Davidovits [4,5] proposed new materials and techniques, named “geopolymer” and was treated as the most promising alternative materials to OPC. Geopolymers, generally made from materials such as metakaolin, fly ash, and ground granulated blast furnace slag (GGBFS), etc., are rich in silicon and aluminum compounds and will react under the stimulation of alkaline solution [6]. These solid wastes can be utilized, reducing CO_2_ emissions by 20–50%, which, to some extent, can protect the environment [7]. In addition, raw materials of geopolymer are mostly by-products of industrial products with abundant reserves and low prices. Compared with OPC, geopolymers have better mechanical, chemical, thermal properties, and durability [6,8,9,10,11,12]. Based on the above-mentioned characteristics, geopolymer is a better option for the development of sustainable products such as building materials, fire-retardant coatings, fiber-reinforced composite materials, and fixed solutions for chemical and nuclear industrial wastes.

However, most previous studies about the alkali-activated fly ash/GGBFS mortar and concrete were carried out by curing samples at elevated temperature or in ambient curing condition [13]. Many researchers believed that addition of GGBFS can significantly improve early and later age mechanical strength of fly ash based geopolymer concrete cured at room temperature [14,15]. Up to now, the research on the physical and mechanical properties of geopolymer concrete curing at negative temperature has not been reported.

**Figure 1:**
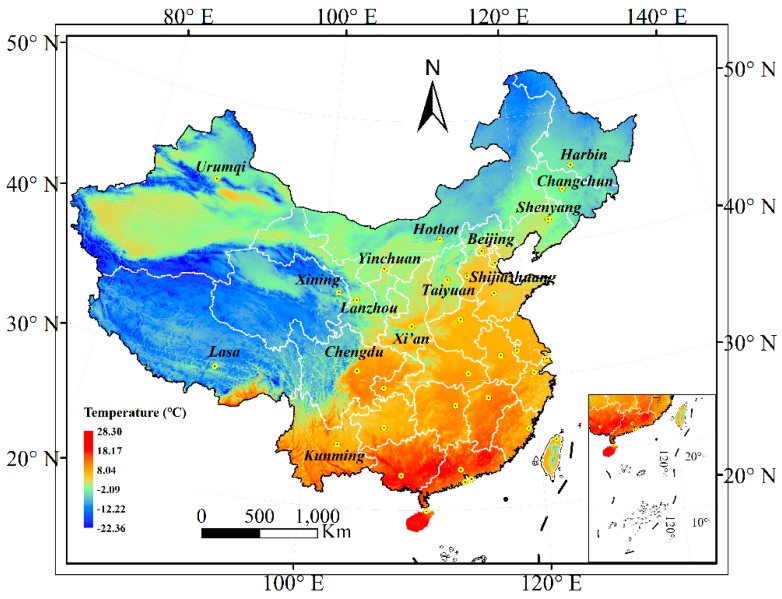
The mean annual air temperature distribution of China in 2015 [16].

With the social development, cleaner and energy-saving building materials are needed in cold and polar regions of the world. For example, Figure 1 shows the mean annual air temperature distribution of China in 2015 [16], and the temperature in most areas is lower than 0 °C, however, many infrastructures were built in these areas, such as Qinghai-Tibet railway, Sichuan-Lasa Expressway, and Harbin Dalian high speed railway. These projects only have a very short construction period of positive temperature (above 0 °C) every year, which will extend the entire construction period and increase the cost. If the in-situ casting geopolymer concrete with better performance can be obtained under the condition of negative temperature curing, the construction period will be shortened and the cost will be saved.

In the OPC hydration process, water is a paramount component participating in chemical reaction to produce hydration products, which makes concrete or mortar have good workability. The role of water in the depolymerization, dissolution, polymerization, and hardening of geopolymer has been explored by some scholars. Weng et al. [17] considered that water was the transport medium of aluminosilicate dissolution, ions transfer, tetrahedral compounds hydrolysis, and tetrahedral units polymerization. Aliabdo et al. [18] revealed that the addition of water improved the workability of mixture, but reduced other properties of geopolymer concrete. The optimum additional water content was 30 kg/m^3^, which has little effect on properties of geopolymer. It was found that using extra water to improve the workability of fly ash geopolymer had higher strength than adding superplasticizer [19]. Literature [20] reported that the lower the initial water content, the higher the strength growth rate of geopolymer cured at room temperature from 7 days to 14 days, in addition, the strength growth rate of heat-cured specimens did not seem to be affected by the lower initial water content. However, hydration process of geopolymer cured at negative temperature will be more complex, water content is still a vital factor.

In view of the above-mentioned background, it is necessary to explore the characteristics of geopolymer cured at negative temperature. In this paper, the influence of water content was investigated to see whether it could be beneficial on improving the properties of fly ash and GBBFS-based geopolymer mortar at negative temperature cured. A series of laboratory tests were carried out by testing the strength, observing the microstructure characteristics and identifying the formation of minerals. −5 °C was selected as a representative curing temperature to simulate weather and construction conditions in cold and polar regions. These characteristics were also compared with the research results of ambient-cured specimens in previous literature.

## 2. Materials and Methods

### 2.1. Raw Materials

**Figure 2:**
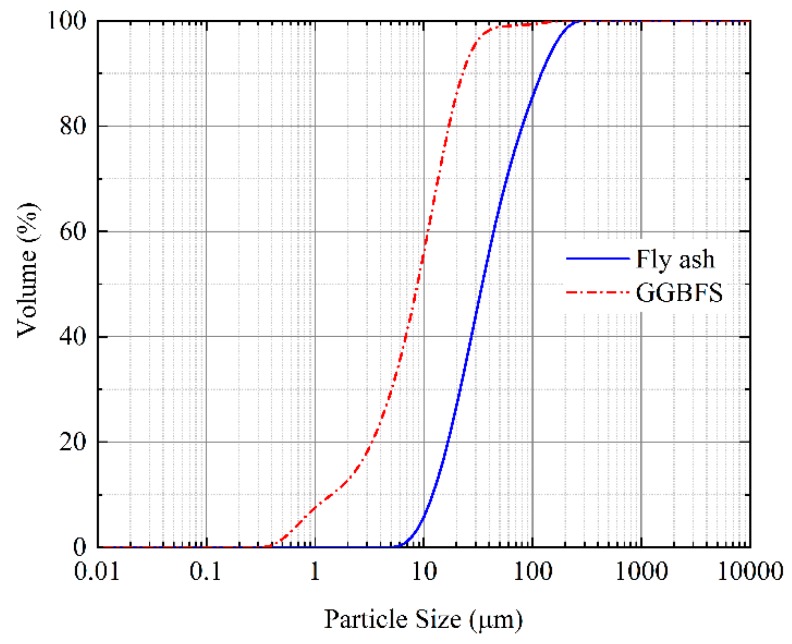
Initial particle distribution curves of FA and GGBFS.

**Figure 3:**
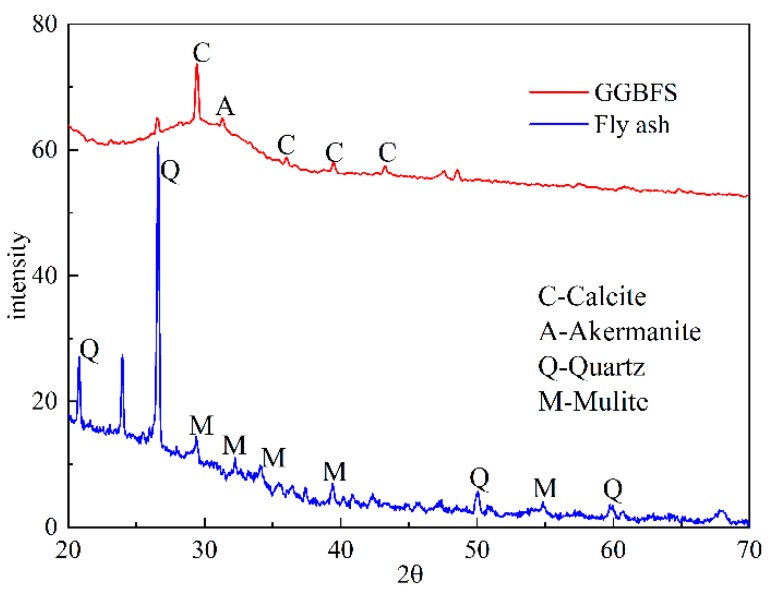
XRD patterns of FA and GGBFS.

In this study, a low calcium fly ash (Class F) was obtained from a thermal power plant in Yulin city, Shanxi province, China. GGBFS was used as the major ingredient [21]. The initial particle distribution curves of the fly ash (FA) and GGBFS are shown in Figure 2, the average particle size is 34.46 μm and 8.75 μm, respectively. The chemical components of FA and GGBFS are shown in Table 1, which were determined by a Bruker S8 TIGER Series 2 X-ray fluorescence (XRF, Bruker Corporation, Karlsruhe, Germany) apparatus. The chemical component of FA is vital important to the test results [22]. Moreover, Figure 3 exhibited XRD patterns of the FA and GGBFS, respectively, which were conducted on a Bruker D8 ADVANCE apparatus. The equipment mentioned above is produced by Bruker company in Germany. XRD patterns clearly showed the presence of quartz (SiO_2_), mullite (3Al_2_O_3_·2SiO_2_), calcite (CaCO_3_), and akermanite (Ca_2_Mg (Si_2_O_7_)) crystalline phase, which were indicated by sharp peaks.

Sodium hydroxide (NaOH) and sodium silicate (Na_2_SiO_3_) solution were selected as alkaline activator after comparing available activators [23].The modulus of sodium silicate was 3.306 with chemical composition of 26.65% SiO_2_, 8.32% Na_2_O, and 65.03% H_2_O. The NaOH solution was formulated with 60 g tap water and 28.80 g NaOH solid particles. The purity of sodium hydroxide pellet was more than 96%. The prepared concentration of sodium hydroxide solution was remained constant 12 mol/L in all mixtures.

The standard sand was obtained from an ISO Standard Sand Co., Ltd. (Xiamen) of China. Sodium tetraborate (Na_2_BO_7_·H_2_O) was used as retarder. The content of analytic pure sodium tetraborate was higher than 99.5%. A polycarboxylate based superplasticizer and an antifreeze agent were used in the samples. These additives were used to improve the workability of geopolymer mortar mixture.

### 2.2. Mixture Proportions

The mixes that used in this paper were designed and listed in Table 2. All mixtures contain the same amount of fly ash, GGBFS, and activator solution, except for the additional water content. The only variable was the additional water content. It needed to be emphasized that the “water content” here refers to the total amount of H_2_O brought from additional water, NaOH solution, and sodium silicate solution. The water content in NaOH solution and sodium silicate solution was 60 g and 78.04 g (120 g × 65.03%), respectively. Superplasticizer 1 g (0.22 wt % of binder), retarder 5 g (1.11 wt % of binder), and antifreeze agent 10 g (2.22 wt % of binder) were added to the mixture as additives, respectively. The addition amount of these additives was the ideal addition amount determined by repeated tests, which can ensure that the mortar has desired workability and the moisture in it was not frozen at temperature of −5 °C.

The parameter “Water-to-Binder Mass Ratio” (W/B) was calculated. W/B ratio and critical molar ratios of all mixtures were shown in Table 3. The Si/Al ratio and Na_2_O/SiO_2_ ratio were the most important parameters in many literatures [4,6,24,25,26]. The Si/Al ratio of each group was 2.75, which meant that the three dimensional silico-aluminate structures produced may be the Poly(sialate-siloxo) type (-Si-O-Al-O-Si-O-) and Poly(sialate-disiloxo) type (-Si-O-Al-O-Si-O-Si-O-) according to previous research results [4,5]. Davidovits believed that these two structures can achieve desired mechanical strength. Molar ratio of H_2_O/Na_2_O was another vital parameter for the change of water content. Generally speaking, on the premise of desired workability of mixtures, reducing water consumption as much as possible was conducive to improving the mechanical properties and durability of geopolymer concrete. The mass ratio of Na_2_SiO_3_ solution to NaOH solution (SS/SH) was 1.35, and alkaline solution constituted 46.4% of the total binder by mass.

### 2.3. Mixing, Specimen Preparation, and Curing

The first step was activator solution preparation. The temperature of sodium hydroxide solution would increase due to the large amount of heat generated when sodium hydroxide solid particles were dissolved in water. Some researches premixed the NaOH solutions until the solution reached room temperature within 24 h [27], and others [28] considered that the activator mixed with sodium silicate and sodium hydroxide powder has more advantages than that in liquid form. In this study, the activator was still in the form of liquid solution, the purpose of this operation was to eliminate the unreliability of test results caused by heat originated from sodium hydroxide particles dissolving. The NaOH solution was premixed until its temperature reached room temperature.

The steel mold consisted of a horizontal groove, which can simultaneously form six specimens of 40 mm × 40 mm × 40 mm in size. Before the mortar was put into the mold, a thin layer of demolding agent was coated on the inner surface of the mold to facilitate demolding and ensure the smooth and intact surface of the samples.

**Figure 4:**
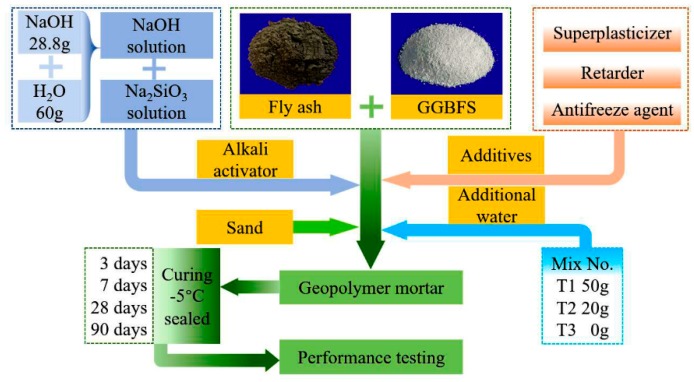
Complete process of samples preparation.

The complete process of samples preparation exhibited in Figure 4. Mortar ingredients were mixed in a laboratory cement mortar mixer (JJ-5). After all the components were accurately weighed, the binders (fly ash and GGBFS) and other dry ingredients were thoroughly mixed in the mixing pan for two minutes. NaOH solution, Na_2_SiO_3_ solution, and additional water were added to the mixture in turn, and then stirred continuously for four minutes. The molds were cast with geopolymer mortars and vibrated compactly on a shaking table. All the above operation steps were carried out at room temperature (about 20 °C). The initial setting of mortar was completed about 45 min. After the vibration compaction, the specimens were packed in a sealed bag and immediately put into a freezer and cured at constant temperature of −5 °C. After 24 h of casting, all specimens were demolded and cured continuously at −5 °C in the freezer until the performance of different ages (3 d, 7 d, 28 d, 90 d) were tested. Owing to the small size of the samples, the temperature of the samples placed into the freezer was lowered to −5 °C in a short time. However, temperature was a vital factor in the chemical reaction of geopolymer. The purpose of this operation process was to simulate the worst situation that geopolymer concrete may encounter on-site casting.

### 2.4. Testing Methods

Three specimens of corresponding ages were taken out from the freezer for uniaxial compressive strength test each time. The uniaxial compressive strength test was carried out on MTS(SANS)-CMT5105 tester (MTS Systems Corporation, Shenzhen, China). The loading rate was set to 0.3 MPa/s and then loaded until the specimen was destroyed [29]. After the uniaxial compressive strength test, the crushed samples were observed for microstructural characteristics using a Scanning Electron Microscope (SEM, FEI Company, Hillsboro, OR, USA). In addition, some crushed samples were taken out for X-ray diffraction (XRD) test. XRD tests were measured on a Bruker D8 ADVANCE diffractometer (Bruker Corporation, Karlsruhe, Germany), 40kV, 40mA, using Cu-Kα radiation. The XRD patterns were recorded within 10°–90° (2θ), with a step size 0.01°, and speed of 0.02°/s.

## 3. Experimental Results and Discussion

### 3.1. Compressive Strength

**Figure 5:**
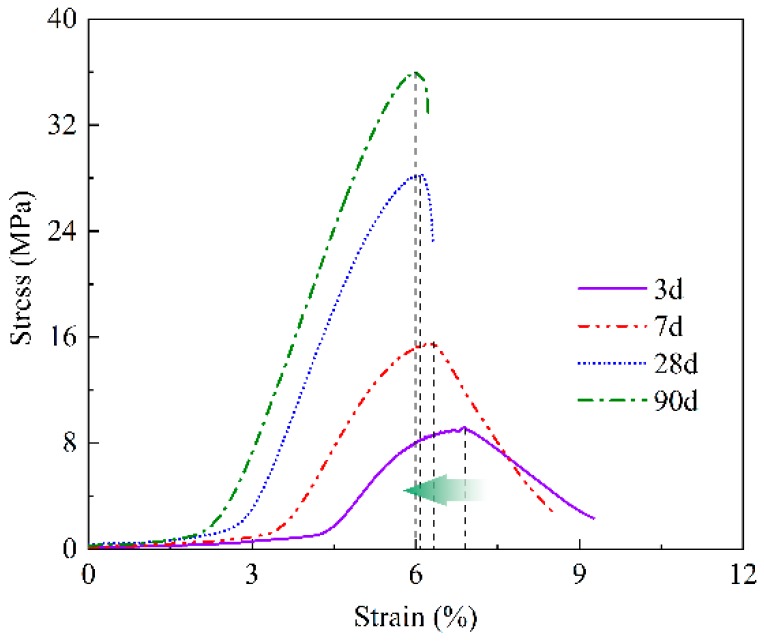
Stress-strain curves of T2 at different ages.

**Figure 6:**
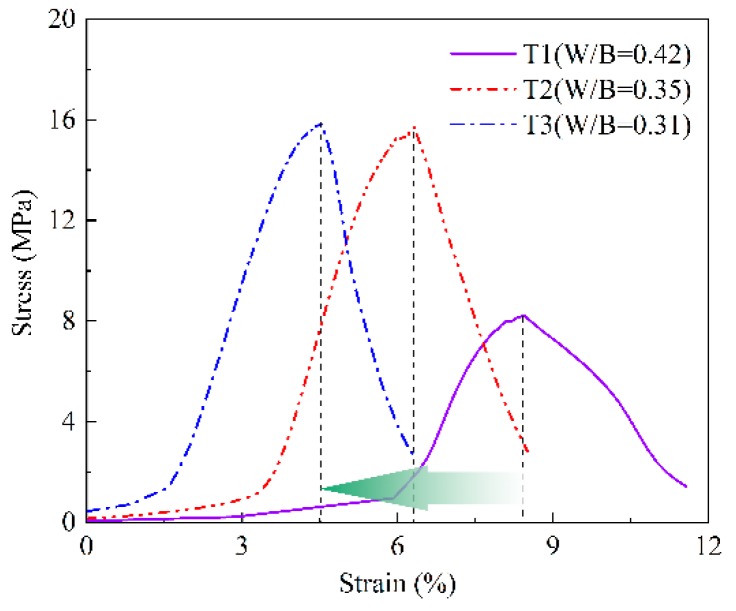
Stress-strain curves of mortars with different Water-to-Binder (W/B) ratios at 7 days.

The compressive strength values of the prepared samples were obtained by uniaxial compression tests. The stress-strain curves of T2 at different ages are plotted in Figure 5. It can be seen that the compressive strength increases and the failure strain decreases gradually with the increasing age. The brittle failure of samples is becoming increasingly evident. T1 and T3 present the same trend, which are not shown repeatedly here. Figure 6 shows the stress-strain curves of each group at age of 7 days. The greater W/B ratio, the lower strength, the failure strain corresponding to peak strength increases gradually, and plastic deformation characteristics become more and more prominent.

**Figure 7:**
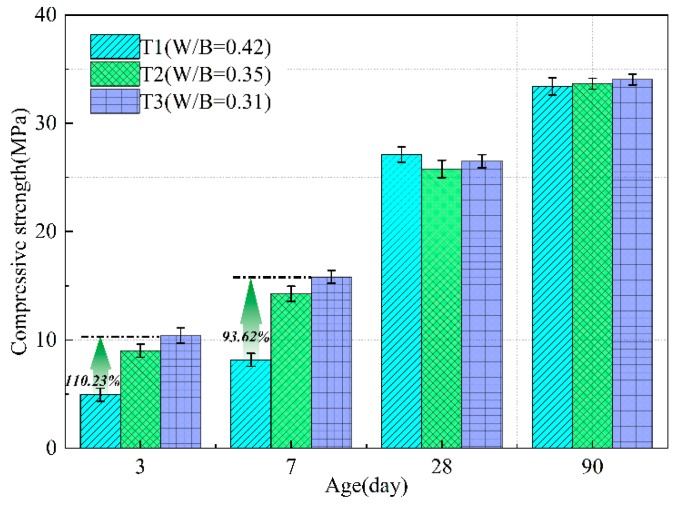
Compressive strengths of mortars with different W/B ratios at different ages.

The compressive strength of mortar samples with different W/B ratios was tested at different ages. Three identical samples were tested each time. The mean values are given in Figure 7. It can be seen that the compressive strength of mortar increases gradually with the decrease of W/B ratio at early age (3 d, 7 d). At the age of 3 days and 7 days, the compressive strength increases by 82.13% and 74.63%, respectively, when the W/B ratio decreases from 0.42 to 0.35; when the W/B ratio decreases from 0.42 to 0.31, it increases by 110.23% and 93.62%, respectively, but when the W/B ratio decreases from 0.35 to 0.31, it increases slightly, 15.43% and 10.87%, respectively.

At the age of 28 days, the compressive strength of each group is basically maintained at the level of 25.78 MPa–27.10 MPa. The compressive strength reached 33.4 MPa–34.04 MPa at the age of 90 days, and the values are very close. It can be concluded that different water content has little influence on long-term strength.

The mechanism of the influence of water content on the strength of geopolymer is discussed based on the above experimental results. It can be clearly observed that, on the prerequisite of satisfying the workability of mortar, reducing the water consumption is beneficial to improving the early strength of mortar under negative temperature conditions. In fact, adding extra different amounts of water has changed the initial molar concentration of NaOH solution, which is no longer a fixed value of 12 mol/L, but less than 12 mol/L. The ultimate concentration of NaOH solution of T1, T2, and T3 was 3.83 mol/L, 4.56 mol/L, and 5.22 mol/L, respectively. The higher the water content is, the smaller the molar concentration is. The addition of extra water changes the excitation ability of the activator. Higher initial molar concentration of NaOH solution promotes the dissolution of aluminosilicate and forms and tetrahedral units in the early age, leading to the augment of strength at the early stage [30]. Within a certain range, the increased NaOH concentration helps to improve the compressive strength of concrete [27,31,32], which is consistent with the results in this paper. However, when the concentration of NaOH solution exceeds a certain limit, the polycondensation process was hindered and the compressive strength would be reduced [33,34]. From the results of this study, the initial concentration of NaOH solution did not exceed the limit value, and the strength value monotonously increased with the increasing concentration.

Moreover, the mechanism of the influence of water content on the strength of geopolymer is discussed from the reaction equation. Davidovits [4,5] summarized the basic principles of the chemical reactions as follows: Equations (1) and (2):

(1) $$\begin{array}{l} {{(\mathrm{Si}}_{2}O_{5}\cdot {\mathrm{Al}}_{2}O_{2})}_{n}+3nH_{2}O\overset{\mathrm{NaOH}/\mathrm{KOH}}{\to }n{\left(\mathrm{OH}\right)}_{3}\text{-Si-O-Al}\overset{\left(\text{-}\right)}{\text{-}}{\left(\mathrm{OH}\right)}_{3}\\ n{\left(\mathrm{OH}\right)}_{3}\text{-Si-O-Al}\overset{\left(\text{-}\right)}{\text{-}}{\left(\mathrm{OH}\right)}_{3}\overset{\mathrm{NaOH}/\mathrm{KOH}}{\to }(\mathrm{Na},K)\text{-}(\text{-}\overset{|}{\underset{\begin{array}{l} \;|\\ O\\ \;| \end{array}}{\mathrm{Si}}}\text{-O-}\overset{|}{\underset{\begin{array}{l} \;|\\ O\\ \;| \end{array}}{\mathrm{Al}}}\overset{\left(\text{-}\right)}{\text{-}}{O\text{-})}_{n}+3nH_{2}O \end{array}$$

(2) $$\begin{array}{l} {{(\mathrm{Si}}_{2}O_{5}\cdot {\mathrm{Al}}_{2}O_{2})}_{n}+n2{\mathrm{SiO}}_{2}+4nH_{2}O\overset{\mathrm{NaOH}/\mathrm{KOH}}{\to }n{\left(\mathrm{OH}\right)}_{3}\text{-Si-O-}\overset{\left(\text{-}\right)}{\underset{\begin{array}{c} |\\ {\left(\mathrm{OH}\right)}_{2} \end{array}}{\mathrm{Al}}}{\text{-O-Si-}(\mathrm{OH})}_{3}\\ n{\left(\mathrm{OH}\right)}_{3}\text{-Si-O-}\overset{\left(\text{-}\right)}{\underset{\begin{array}{c} |\\ {\left(\mathrm{OH}\right)}_{2} \end{array}}{\mathrm{Al}}}{\text{-O-Si-}(\mathrm{OH})}_{3}\overset{\mathrm{NaOH}/\mathrm{KOH}}{\to }(\mathrm{Na},K)\text{-}(\text{-}\overset{|}{\underset{\begin{array}{l} \;|\\ O\\ \;| \end{array}}{\mathrm{Si}}}\text{-O-}\overset{|}{\underset{\begin{array}{l} \;|\\ O\\ \;| \end{array}}{\mathrm{Al}}}\overset{\left(\text{-}\right)}{\text{-}}O\text{-}\overset{|}{\underset{\begin{array}{l} \;|\\ O\\ \;| \end{array}}{\mathrm{Si}}}{\text{-}O)}_{n}+4nH_{2}O \end{array}$$

On the one hand, NaOH is a necessary condition for the reaction. The mass of NaOH solid particles is constant in the solution, therefore, the final amount of dissolved aluminosilicate is certain and the amount of geopolymer products is certain. On the other hand, it can be seen from the reaction process that the water molecules on the left and right sides of the reaction equation are balanced. Therefore, the study [18] believed that water had no role in the chemical reaction except for providing workability. From the reaction equation alone, the content of water does not affect the amount of geopolymer products. These two aspects are the reason why the long-term strength is very close.

### 3.2. Microstructural Properties

#### 3.2.1. SEM Observation

**Figure 8:**
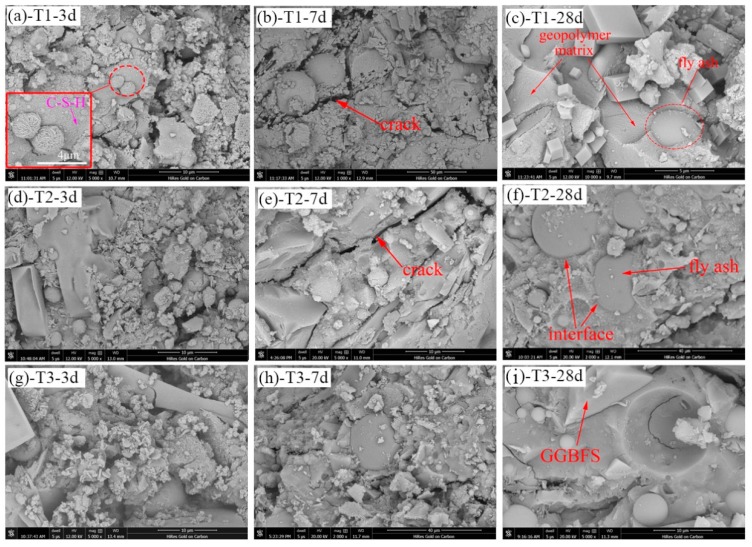
Micrographs of samples at different ages: (**a**,**d**,**g**) 3 days; (**b**,**e**,**h**) 7 days; (**c**,**f**,**i**) 28 days.

In this section, the influence of water content on the microstructure of geopolymer is discussed. The micrographs of samples have been obtained using SEM as shown in Figure 8a–i. All images clearly show the presence of fly ash cenosphere and slag particles that have completely or partially undergone geopolymer reactions and been embedded in a continuous matrix. The usual trend is that with the same water content, the structure becomes more and more dense for increasing of age.

At an age of 3 days, the unreacted fly ash particles in Figure 8d,g are significantly less than those in Figure 8a. As shown in Figure 8a, after enlarging 10,000 times, the image clearly indicates that the surface of fly ash microspheres is disintegrated and the mullite skeleton is exposed under the excitation of alkaline activator, and the presence of C-S-H gel can also be observed in the diagram. At an age of 7 days, the pores and cracks in Figure 8e,h are obviously less than those in Figure 8b, generating a large number of geopolymer matrix, and the structure shows more compactness. At an age of 28 days, the specimens formed a dense amorphous-gel structure (Figure 8c,f,i), some unreacted FA and GGBFS particles were still immersed in the gel, the interface between particles and gel is very tight, and there was no difference in the three plots.

It can be concluded that water content has a certain influence on the microstructure of geopolymer in early age. The smaller the water content is, the less unreacted components are, the denser the structure is and the higher the strength is. In addition, the long-term microstructure of geopolymer is not affected by water content. This will explain in terms of microstructure why the geopolymer mortar has low water content but high strength at early age. At the same time, the reason why the strength increases with curing time is explained.

#### 3.2.2. XRD Analysis

**Figure 9:**
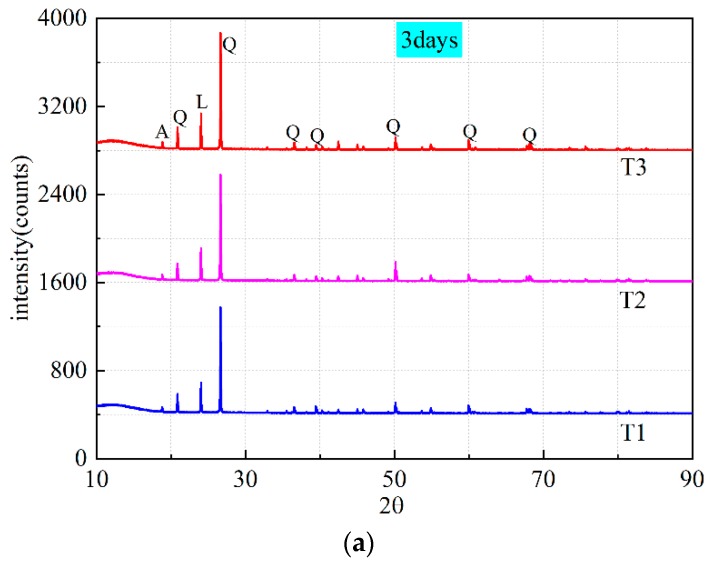

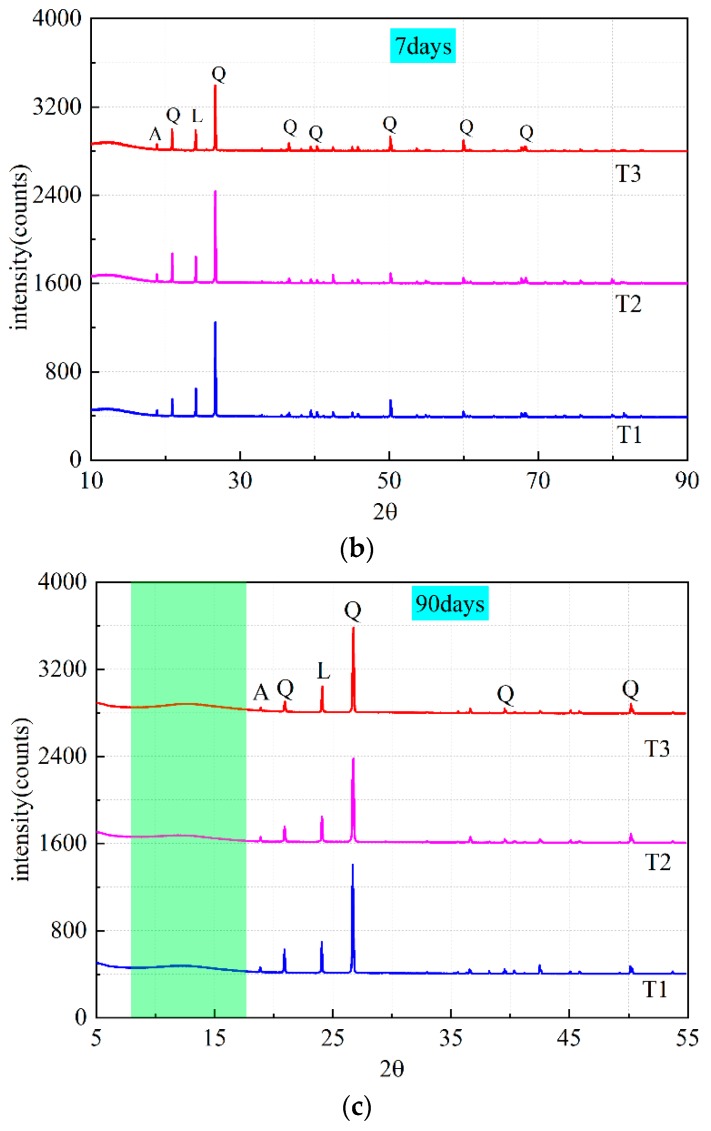
XRD patterns of samples (T1, T2, T3) at different ages (Q-quartz, A-amarillite, L-lazurite): (**a**) 3 days; (**b**) 7 days; (**c**) 90 days.

XRD patterns for all mixtures at different ages are shown in Figure 9a–c. Presence of quartz, lazurite, and amarillite (NaFe^3+^(SO_4_)_2_·6H_2_O) can be observed in all samples and the patterns are quite similar. This shows that different water content only affects the hydration process, and the final hydration products are the same.

The complex composition of lazurite ((Na, Ca, K)_7–8_Si_6_Al_6_O_24_(SO_4_, S, Cl)2·nH_2_O) belongs to the sodalite group [35], and the molecular structure indicates the formation of sodium aluminosilicate hydrate(N-A-S-H) gel and calcium aluminosilicate hydrate (C-A-S-H) gel. Sodalite framework is a typical geopolymeric structure [5]. Lazurite mineral crystal has zeolite-like structure. The trisulfur (S^3−^) is trapped into a cage of the zeolitic sodalite, which results in the sample manifesting a blue or green color [36,37]. The studies [36,37] showed that the specific color of exterior surface turned to “gray” or “white” when the concrete or mortar contacted with air. This conclusion is proved by the fact that the surface color of the damaged specimen is grey and the interior is green.

A broad hump between 10° and 20° (*2θ*) can be observed on the XRD patterns in Figure 9c, which usually represents the existence of amorphous phase. Compared with geopolymer cured at room temperature [38], the hump height is lower. The lower hump height means that the amorphous gel generated by the geopolymerization reaction is less. It can be drawn a conclusion that the curing temperature of geopolymer is an extremely crucial factor. Negative temperature curing reduces the reaction degree of geopolymer.

### 3.3. Elasticity Modulus

The static elastic modulus ($E_{c}$) of concrete is a very important performance index in structural design. Generally, the slope of the stress-strain curve at the elastic stage represents the elastic modulus of the material. The compressive strength ($f_{c}$) and modulus of elasticity increase with age (Figure 5). It can also be observed in Figure 6, the elastic modulus of T1 is lower than that of T2 and T3, and T2 and T3 are close to each other. In other words, the modulus of elasticity increases with the decrease of W/B ratio at the same age. 

The modulus of elasticity is closely related to compressive strength. Some experimental values and predictive formulas have been given in previous studies on geopolymer [39,40,41,42]. Most of the results were acquired from experiments with heat-cured geopolymer concrete. However, no result is currently available for negative temperature cured geopolymer. Therefore, it is essential to explore the prediction model of geopolymer under negative temperature curing. To evaluate the elastic modulus of geopolymer mortar in this study, different existing codes and equations have been proposed, as described below.

American Concrete Institute: According to the ACI Building Code Requirements for Structural Concrete ACI 318-14 [43], elastic modulus of concrete can be calculated by Equation (3):

(3) $$E_{c}=0.043\times \rho^{1.5}\times \sqrt{f_{c}^{\prime }}$$

where $E_{c}$ is the modulus of elasticity; $f_{c}^{\prime }$ is the specified compressive strength (MPa) of concrete after 28 days of curing; ρ is density of concrete ranging from 1442 to 2483 kg/m^3^, it takes the measured average value 2404 kg/m^3^ in this study; the relationship between the measured compressive strength $f_{cm}$ and $f_{c}^{\prime }$ are given by Equations (4)–(6) [44].

(4) $$f_{cm}=f_{c}^{\prime }+7.0\;for\;f_{c}^{\prime }\leq 21\;MPa$$

(5) $$f_{cm}=f_{c}^{\prime }+8.3\;for\;21<f_{c}^{\prime }\leq 35\;MPa$$

(6) $$f_{cm}=1.1f_{c}^{\prime }+5.0\;for\;f_{c}^{\prime }>35\;MPa$$

CEB-FIP Model Code: The elastic modulus of concrete can be estimated by Equation (7) [45].

(7) Ec=0.85×2.15×104×(fc10)13

where $f_{c}$ is the mean measured value of compressive strength (MPa).

Hardjito et al. [42]. Recommended a practical formula expressed by Equation (8) to predict elastic modulus of fly ash based geopolymer concrete cured at high temperatures.

(8) $$E_{c}=2707\times \sqrt{f_{c}^{\prime }}+5300$$

In another article [40], Nath and Sarker put forward Equation (9) to predict the elastic modulus of geopolymer concrete cured at room temperature.

(9) $$E_{c}=3510\times \sqrt{f_{c}}$$

Lee and Lee [46] proposed a prediction formula for the elastic modulus of fly ash/slag based geopolymer concrete, as exhibited below.

(10) $$E_{c}=5300\times \sqrt[{3}]{f_{c}}$$

**Figure 10:**
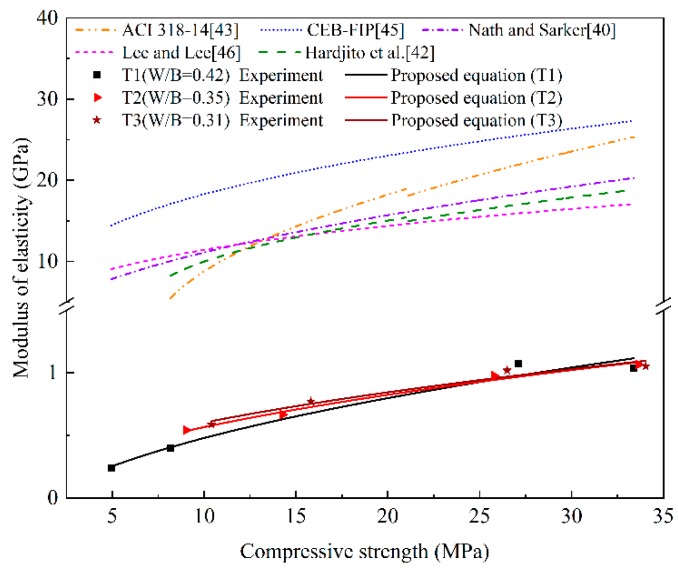
Comparison of tested and predicted results.

**Figure 11:**
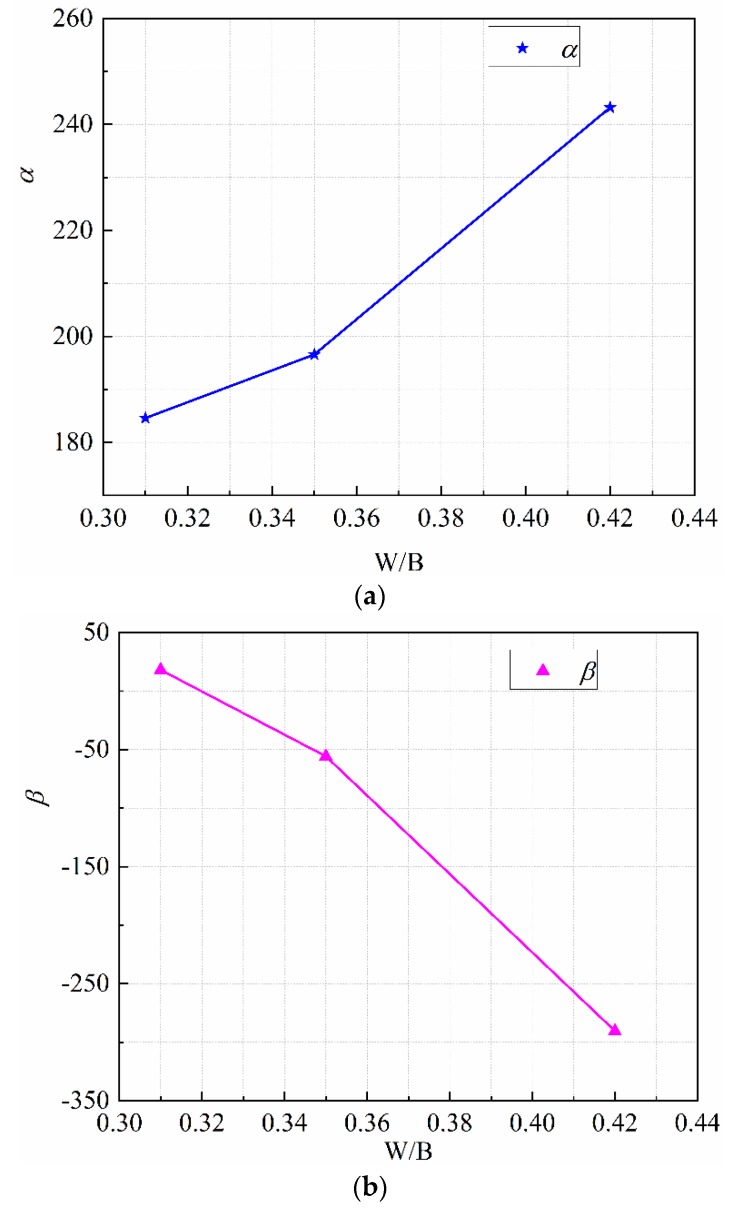
Variation of coefficients α and β with water binder ratio: (**a**) α; (**b**) β.

The values of elastic modulus at different ages (3 d, 7 d, 28 d, 90 d) for geopolymer mortar were obtained from the stress-strain curves. Each elastic modulus value is the mean of three identical samples with a standard deviation less than 12 MPa. The values are drawn in Figure 10 and compared with predicted values using above models. In the figure, both the experimental and predicted values exhibited the general trend of increasing elastic modulus for increasing geopolymer mortar compressive strength. It is clear that, the tests values are lower than any predicted values, the difference is grate. This result is attributed to the applicability of the recommended equations and model codes. The formulas of ACI 318-14 and CEB-FIP code are intended for original Portland concrete (OPC). The equations in references [40,42,46], are applicable to estimate geopolymer concrete cured at elevated temperature or in ambient condition. Moreover, some studies [41,47] have reported that the elastic modulus of OPC is slightly higher than geopolymer concrete. However, in this study, the geopolymer mortar specimens were cured at temperature of −5 °C, these existing equations are no longer applicable. Based on the analysis of the above formulas and test values, a new empirical model is proposed. The square root form of compressive strength ($\sqrt{f_{c}}$) is still used. The least square method is used for regression analysis of data in a given equation, and the ultimate equation is shown as follows.

(11) $$E_{c}=\alpha \times \sqrt{f_{c}}+\beta$$

where $f_{c}$ is compressive strength of geopolymer mortar (MPa). Results indicated that the coefficients *α* and *β* in the model equation are not fixed values, but varying with W/B ratios, which are listed in Table 4 and drawn in Figure 11. It can be observed that the coefficients *α* increases with the increasing of W/B ratio, while the coefficient *β* decreases with the increasing of W/B ratio. In additional, the correlation coefficient *R* of different W/B ratios is very high. Hence, Equation (11) is applicable to predict the elastic modulus of geopolymer mortar cured under negative temperature. The coefficients *α* and *β* can be selected by interpolation according to the W/B ratio.

## 4. Conclusions

The influence of water content on the properties of fly ash and GBBFS-based geopolymer mortars cured at −5 °C was investigated. Several interesting conclusions can be drawn from the results presented thus far:

(1) Water content has an impact on compressive strength of geopolymer mortar at the age of 3 days and 7 days. At the age of 28 days, the compressive strength of each group is basically maintained at the level of 25.78 MPa–27.10 MPa. The compressive strength reached about 33.4 MPa at the age of 90 days.
(2) Higher molar concentration of NaOH solution promotes the dissolution of aluminosilicate in early age, which leads to the increase of strength in early stage. Lower water content is beneficial to improving the early strength of mortar under negative temperature conditions, however, water content has little effect on long-term strength.
(3) An empirical model is proposed to predict $E_{c}$, which exhibited better applicability for geopolymer mortar cured at temperature of −5 °C. The change of water content affects the coefficients in the model.

## Figures and Tables

**Table 1:** Chemical components of fly ash (FA) and ground granulated blast furnace slag (GGBFS) by X-ray fluorescence (XRF) analysis.

Chemical Composition (wt %)	Fly Ash	GGBFS
SiO_2_	51.70	28.57
Al_2_O_3_	15.68	13.55
CaO	9.96	30.44
Fe_2_O_3_	19.06	2.92
MgO	1.48	9.80
Na_2_O	0.57	0.59
K_2_O	1.04	0.34
S	0.27	0
other	0.24	13.79

**Table 2:** Details of geopolymer mortar mix proportions.

Mix No.	Mortar Mixture Quantity (Unit: g)								
	Fly Ash	GGBFS	Additional Water	Sand	SH ^1^	SS ^2^	SP ^3^	BR ^4^	AF ^5^
T1	225	225	50	1350	88.8	120	1	5	10
T2	225	225	20	1350	88.8	120	1	5	10
T3	225	225	0	1350	88.8	120	1	5	10

^1^ NaOH solution; ^2^ Na_2_SiO_3_ solution; ^3^ Superplasticizer; ^4^ Borax; ^5^ Antifreeze agent.

**Table 3:** Critical molar ratios and water/binder of all mixtures.

Mix No.	Water/Binder	Molar Ratio		
		Si/Al	Na_2_O/SiO_2_	H_2_O/Na_2_O
T1	0.42	2.75	0.09	51.44
T2	0.35	2.75	0.09	43.23
T3	0.31	2.75	0.09	37.76

**Table 4:** Equation Parameters of the empirical Model.

Mix No.	W/B	Equation (11)		
		α	β	R
T1	0.42	260	−335	0.97
T2	0.35	197	−56	0.98
T3	0.31	185	18	0.96
